# Hemozoin induces neuronal injury primarily characterized by axon rupture and mitochondrial damage in experimental cerebral malaria

**DOI:** 10.1186/s13071-025-07102-5

**Published:** 2025-12-18

**Authors:** Tong Li, Dongmei Dang, Yan Shen, Jun Wang, Yuxiao Huang, Qinghao Zhu, Yi Wang, Chao Yang, Ganze Li, Jiayi Sun, Aining Zhang, Pengtao Li, Jiao Liang, Ya Zhao

**Affiliations:** 1 School of Medical, Yan’an University, Yan’an, 716000 Shaanxi China; 2 Department of Medical Microbiology and Parasitology, Air Force Medical University, Xi’an, 710032 Shaanxi China; 3 Pathogenic Biology Teaching and Research Office, Yan’an University, Yan’an, 716000 Shaanxi China; 4 College of Life Sciences, Northwest University, Xi’an, 710069 Shaanxi China; 5 School of Medicine, Northwest University, Xi’an, 710069 Shaanxi China

**Keywords:** Cerebral malaria, Hemozoin, Neuron, Mitochondria

## Abstract

**Background:**

Cerebral malaria (CM) is the most serious and fatal neurological complication of *Plasmodium falciparum* infection, which can cause death or long-term neurological sequelae. Neuronal injury is a primary cause of these sequelae in patients with CM; however, the underlying mechanisms remain incompletely elucidated. Hemozoin (Hz), the metabolic byproduct of hemoglobin digested by *Plasmodium* parasites, is closely associated with the severity of CM. However, it is not clear whether Hz is a direct contributor to neuronal injury.

**Methods:**

C57BL/6 J mice were infected with the *Plasmodium berghei* ANKA (PbA) strain to induce experimental cerebral malaria (ECM). Hz deposition and neuronal injury in ECM mice brain tissues were assessed using histopathological staining. In vitro, primary cortical neurons were stimulated with purified hemozoin (pHz). Neuronal morphology, pHz internalization, and injury severity were assessed via transmission electron microscopy (TEM), live-cell imaging, and lactate dehydrogenase (LDH) assays, respectively. Furthermore, Mito-Tracker and JC-1 probes were used to analyze mitochondrial content and membrane potential, respectively. ATP assay kits were used to quantify cellular energy metabolism levels, while reactive oxygen species (ROS)/neuronal nitric oxide synthase (nNOS) fluorescent probes were used to assess oxidative stress and inflammatory response. Neurotransmitter alterations were analyzed by measuring glutamate (Glu) levels.

**Results:**

In the cerebral cortex of ECM mice, significant Hz deposition and reduced neuronal nuclei (NeuN) expression levels were observed. Immunofluorescence (IF) staining demonstrated that pHz adhered to primary neurons in vitro, causing reduced dendritic arborization, axon rupture, and plasma membrane disruption. TEM and live-cell imaging confirmed that pHz was internalized into the cytoplasm of neurons. Furthermore, pHz induced mitochondrial structural damage and reduced mitochondrial content. Concurrently, pHz triggered mitochondrial dysfunction, characterized by diminished mitochondrial membrane potential (MMP), reduced ATP levels, and elevated ROS. In addition, pHz upregulated intraneuronal nNOS activity and caused a decrease in neurotransmitter levels.

**Conclusions:**

This study provided the first evidence to our knowledge that Hz directly adhered to neurons and underwent internalization into its cytoplasm, thereby leading to neuronal injury. These findings elucidate a potential mechanism underlying neuronal injury in ECM and inform the development of adjuvant therapies targeting Hz.

**Graphical Abstract:**

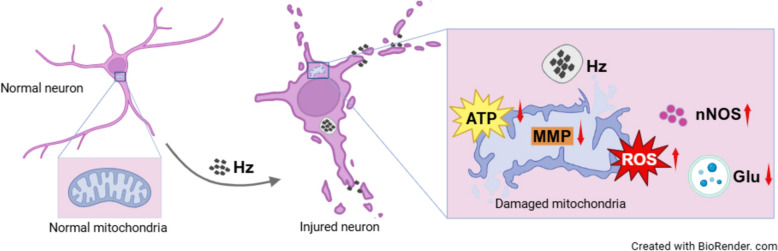

**Supplementary Information:**

The online version contains supplementary material available at 10.1186/s13071-025-07102-5.

## Background

Malaria, caused by *Plasmodium* ​infection, inflicts a significant global health burden and was responsible for ​​an estimated 263 million malaria cases and 597,000 deaths in 2023 ​​[1]. Cerebral malaria (CM)​​, the most severe and fatal neurological complication ​​of​​ *Plasmodium falciparum* infection [2, 3], ​​manifests as​​ coma, hemiplegia, convulsions, ataxia, impaired consciousness, and ​​ultimately death​​, ​​with most cases occurring in African children under 5 years of age. The current pathogenesis of CM involves the sequestration of parasitized red blood cells (pRBCs) in the cerebral microvascular endothelium, which synergizes with immune cell infiltration to induce immunopathological damage, thereby disrupting the blood-brain barrier (BBB) [4]. The resultant cerebral microhemorrhages facilitate the extravasation of inflammatory mediators and parasite-derived metabolites into the brain parenchyma, ultimately causing neuronal injury​​ and ​​long-term neurological sequelae ​​[5–7]. Clinical studies​​ have shown that ​​pediatric​​ CM patients ​​exhibit​​ more severe inflammatory responses, ​​cerebral​​ hemorrhages, and neuronal injury ​​than non-CM patients [8, 9]​​. ​​Critically, neuronal axonal injury ​​is strongly associated with​​ persistent neurological deficits and cognitive impairment [10]. However, ​​the mechanisms underlying neuronal injury remain incompletely elucidated, hindering the development of targeted adjuvant therapies.

Our previous studies have demonstrated that activated CD8^+^ T cells play a role in inducing neuronal injury in the pathogenesis of ECM [11, 12]. However, it remains unclear whether additional factors directly mediate neuronal injury​​. During intraerythrocytic proliferation, *Plasmodium* parasites release substantial quantities of parasitic proteins, lipids, nucleic acids, and metabolites into the circulation upon rupture of pRBCs [13]. Hemozoin (Hz), the byproduct of hemoglobin digestion by *Plasmodium* parasites, is one of the most important metabolites released into the blood circulation, which infiltrates the brain parenchyma following BBB disruption, and probably persists in brain tissue long after parasite clearance, causing prolonged damage to brain cells [14, 15].

Several studies have established a close relationship between Hz deposition and CM severity [14, 16]. Postmortem analysis​​ of pediatric CM brain tissues revealed significantly more extracellular Hz deposits, ​​along with​​ sequestered hemozoin-containing pRBCs, than in specimens from patients with severe malarial anemia (SMA) or non-malarial encephalopathy [17]. Experimental cerebral malaria (ECM) mice infected with the lethal *Plasmodium berghei* NK65 strain deposited significantly more Hz in brains than those infected with the non-lethal *Plasmodium chabaudi* AS strain [18]. Recent research shows that Hz induced apoptosis in co-cultured neurons and astrocytes [19]. Additionally, Hz-activated microglia exacerbated neurotoxicity by promoting pro-inflammatory cytokine expression.​ Treatment with synthetic hemozoin (sHz) induced significant cytotoxicity in differentiated neural progenitor cells (ReNcell VM) [20]. However, whether Hz directly induces neuronal injury and the underlying mechanisms remain unclear.

This study investigated the direct neurotoxic effects of ​​Hz​​ and preliminarily elucidated​​ the underlying mechanisms of neuronal injury during ECM pathogenesis, ​​thereby facilitating the development of adjuvant neuroprotective therapies, potentially mitigating the neurological sequelae of CM.

## Methods

### Reagents and antibodies

The primary antibodies against mouse neuronal nuclei (NeuN, 66836-1-Ig) and microtubule-associated protein 2 (MAP2, 67015-1-Ig) were purchased from Proteintech (China). A fluorescently labeled secondary antibody, FITC-conjugated goat anti-mouse IgG (GB22301), used in immunofluorescence (IF) staining, was sourced from ServiceBio (China).

### Induction of experimental cerebral malaria

Four-week-old male C57BL/6 mice were purchased from the Animal Center of Fourth Military Medical University and housed in specific pathogen-free (SPF) conditions with a 12-h light/dark cycle. The *Plasmodium berghei* ANKA (PbA) strain was maintained in our laboratory. All mice were randomly assigned to different groups, and experimental mice were infected intraperitoneally (i.p.) with 1 × 10^6^ pRBCs to induce ECM, as previously reported by our group [21]. On day 7 post-infection (dpi), mice were killed humanely, and brain tissues were collected for analysis.

### Isolation and culture of primary cortical neuron

Brains were immediately collected from newborn mice within 24 h of birth.​​ Meninges were removed, and tissues were washed with ice-cold Hanks' Balanced Salt Solution (HBSS), minced, and digested with 0.25% trypsin-EDTA (25200-056, Gibco, USA) at 37 °C for 10 min. Digestion was neutralized with three volumes of Neurobasal-A medium (10888–022, Gibco, USA) supplemented with 10% FBS (16140063, Gibco, USA). Tissues were triturated into single cells, centrifuged, and resuspended in Neurobasal-A medium supplemented with 2 mM l-glutamine (25030-164, Gibco, USA), 2% B-27 (17504-044, Gibco, USA), and 1% penicillin-streptomycin (14140-148, Gibco, USA). Cells were plated on poly-d-lysine (A38904-01, Gibco, USA)-coated plates and incubated at 37 °C with 5% CO₂. Half-medium changes were performed every 3 days.

### Hemozoin isolation and quantitation

C57BL/6 mice infected with PbA were killed at 7 dpi. Spleens were collected, mechanically disrupted, and resuspended in 1 mg/ml proteinase K solution. The mixture was incubated at 37 °C for 16 h. The mixture was centrifuged at 11,000 × *g* for 15 min to pellet Hz. The supernatant was removed, and the pellet was washed with deionized water (centrifugation as above). The pellet was then washed with 2% SDS/100 mM NaHCO₃, sonicated for 1 min (10 W, pulse 0.5 s), and centrifuged, and this process was repeated three times. Subsequently, the pellet was resuspended in deionized water. The resulting product was the purified hemozoin (pHz) utilized in this experiment, and all subsequent experiments were conducted using this material. Then, a portion of the pHz was dissolved in 2% SDS/20 mM NaOH for 1 h, and its absorbance at 405 nm was measured for quantification [18]. Neurons cultured in plates were stimulated with pHz (5–40 μg/ml per well). Alternatively, pHz was added at 10 μg/ml per well.

### Transmission electron microscopy (TEM)

Primary neurons were seeded into six-well plates and stimulated with pHz for 48 h. Cells were washed thoroughly with PBS, fixed with pre-chilled 2.5% glutaraldehyde overnight, and post-fixed in 1% osmium tetroxide for 1 h. After PBS washing, samples were progressively dehydrated in graded ethanol solutions and embedded in the Epon 812/DDSA/NMA/DMP-30 mixture. Neuronal ultrastructure was analyzed via TEM (HT7800, HITACHI, Japan) by Servicebio Co., Ltd. (China).

### Scanning electron microscopy (SEM)

pHz-prepared smears were post-fixed in 1% osmium tetroxide for 1 h, washed with PBS, and dehydrated in a graded ethanol series. Samples underwent critical point drying, followed by gold sputter-coating. The specimens were observed under a scanning electron microscope (SEM; Regulus 8100, HITACHI, Japan) by Servicebio Co., Ltd (China).

### Live-cell imaging

Live holotomography was performed​​ using a 3D Cell Explorer microscope (Nanolive SA, Switzerland). Primary neurons were cultured in 35-mm glass-bottom μ-Dishes (Ibidi GmbH, Germany) and maintained under physiological conditions (37 °C, 5% CO₂) using an Ibidi temperature control system. ​​After a 2-min equilibration period, cells were stimulated with pHz, and imaging was initiated​​ immediately.​​ Sequential holographic tomograms were acquired at 3-min intervals over a minimum duration of 16 h. The ​​microscope data were​​ collected and analyzed using ​​STEVE software​​.

### Hematoxylin and eosin (H&E) staining

Mice were transcardially perfused with saline, and brains were fixed overnight in 4% paraformaldehyde (PFA) at 4 °C. Tissues were dehydrated through graded ethanol, cleared, embedded in paraffin, and sectioned at 5-μm coronal slices that were mounted on slides. Sections were deparaffinized, rehydrated, and stained with hematoxylin (10–15 min), differentiated in 1% acidic alcohol, blued in 0.6% ammonium hydroxide, rinsed, and counterstained with eosin for 1–3 min. Slides were dehydrated, cleared, and mounted with neutral balsam.

### *Prussian blue staining​*​

Paraffin-embedded mouse brain sections were prepared as previously described. Slides were incubated in Prussian blue staining solution for 1 h, rinsed with distilled water, and counterstained with 0.1% Nuclear Fast Red for 3–5 min. Sections were rinsed with tap water until the background was cleared, dehydrated, cleared, and mounted with neutral balsam.

### Immunofluorescent (IF) staining

Paraffin-embedded mouse brain sections were prepared as previously described. Coronal sections (5 μm) underwent heat-induced epitope retrieval in citrate buffer, followed by 3% H₂O₂ treatment, blocking with 3% BSA/2% bovine serum/0.2% Triton X-100 in PBS for 30 min, primary antibody incubation at 4 °C overnight, secondary antibody incubation at room temperature for 1 h, and DAPI staining (G1021, Servicebio) for 10 min. Cultured neurons on poly-d-lysine-coated coverslips were fixed in 4% PFA for 20 min, blocked with the same buffer for 30 min, incubated with primary antibodies at 4 °C overnight, and incubated with secondary antibodies for 2 h before mounting in DAPI-containing medium (ab104139, Abcam). Digital slides were scanned using the Pannoramic DESK scanner (P-MIDI, P25, Japan), acquired using CaseViewer 2.4 (3DHISTECH, Hungary), and analyzed with ImageJ.

### LDH release test

Culture supernatants were collected from primary cortical neurons ​​following a 48 h treatment with graded doses of pHz and​​ were subjected to ultracentrifugation (LXP100, Beckman, USA) at 4 °C and 100,000 × *g*​​ for 2 h to ensure complete removal of pHz. Neuronal injury ​​was assessed​​ using a​​ lactate dehydrogenase (LDH) Assay Kit (C0016, Beyotime, China). The ​​absorbance at 490 nm was measured​​ using a ​​microplate reader (Model 680, Bio-Rad, USA).

### Mitochondrial assay

Primary neurons cultured on poly-d-lysine-coated coverslips were stimulated with graded doses of pHz for 48 h. Cells were washed twice with PBS and then stained with Mito-Tracker Red CMXRos (C1035, Beyotime, China) solution at 37 °C for 30 min. After washing with PBS, neurons were fixed in 4% PFA for 15 min. Mitochondrial fluorescence intensity was quantified using confocal microscopy (FV3000, OLYMPUS, Japan).

### Mitochondrial membrane potential (MMP) measurement

Primary neurons were cultured in poly-d-lysine-coated 96-well plates. After 48 h of stimulation with graded doses of pHz, mitochondrial membrane potential (MMP) was assessed using the JC-1 kit (HY-15534, MCE, USA). Neurons were incubated with 2 μM JC-1 working solution at 37 °C for 20 min, and the fluorescence intensity was measured using a microplate reader (SpectraMax iD3, Molecular Devices, USA). According to the manufacturer's instructions, measurements were recorded for red fluorescence (Ex = 585 nm, Em = 590 nm) and green fluorescence (Ex = 510 nm, Em = 527 nm), respectively.

### Intracellular ROS and nNOS measurement

Primary neurons were cultured on poly-d-lysine-coated coverslips as previously described. Following 48 h of pHz stimulation, intracellular reactive oxygen species (ROS) and neuronal nitric oxide synthase (nNOS) levels were quantified with the ROS Assay Kit (S0033S, Beyotime, China) and NOS Assay Kit (S0025, Beyotime, China), respectively. Neurons were incubated with detection probes for 30 min at 37 °C and washed twice with PBS, and the fluorescence intensity was measured using fluorescence microscopy (BX51, OLYMPUS, Japan).

### Glutamate content detection

Glutamate (Glu) levels were quantified using the Glutamate Content Assay Kit (BC1585, Solarbio, China). Primary neurons were seeded on poly-d-lysine-coated coverslips using a previously described protocol. Neurons were stimulated with pHz for varying durations and washed twice with PBS, and the assay was performed according to the manufacturer's instructions. The ​​absorbance at 340 nm was measured​​ using a ​​microplate reader (Model 680, Bio-Rad, USA).

### ATP quantification

Primary neurons were seeded onto poly-d-lysine-coated coverslips using a previously described protocol, ​​stimulated with graded doses of pHz​​ for 48 h, and their cell lysates were centrifuged at 12,000 × *g* for 10 min at 4 °C. ​​Subsequently​​, the ATP Assay Kit (S0027, Beyotime, China) was applied per the manufacturer's instructions. ATP levels were quantified with a microplate reader (SpectraMax iD3, Molecular Devices, USA).

### Statistical analysis

Data were processed and analyzed with GraphPad Prism software 9.0. Quantitative data were expressed as mean ± SD. Two-group comparisons were performed with Student’s t-tests. Normality tests were performed before t-tests, and the sample data followed a normal distribution. Statistical significance was set at *P* < 0.05.

## Results

### Hz deposition correlates with neuronal injury in the ECM brain

**Fig. 1:**
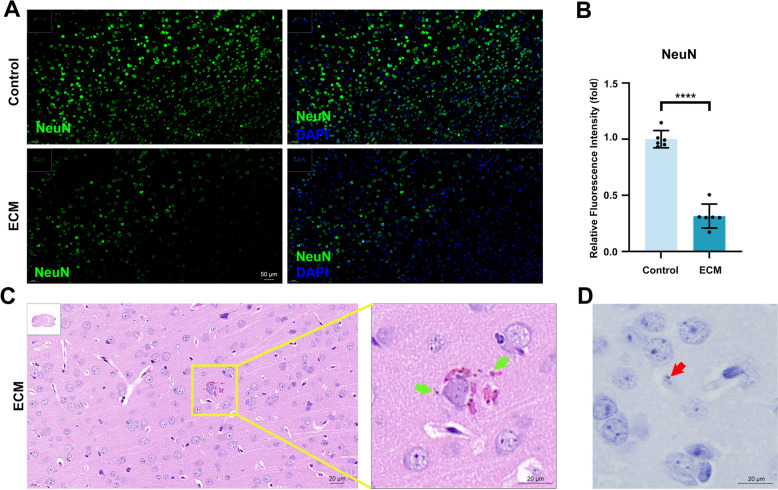
Hz deposition and neuronal injury in the ECM mice brain. **A** IF staining of neurons (NeuN^+^) in the cerebral cortex of control and ECM mice. **B** Statistical graph of relative fluorescence intensity of NeuN in (**A**). Data are expressed as mean ± SD; unpaired t-test; *n* = 6 fields per group. **C** H&E staining of cerebral cortical tissue sections from ECM mice (green arrows: Hz).** D** Prussian blue staining of ECM mouse cerebral cortex tissue sections (red arrows: Hz). ****p < 0.0001. IF: immunofluorescent. ECM: experimental cerebral malaria. NeuN: neuronal nuclei

To investigate the potential relationship between Hz deposition and neuronal injury in ECM, we first generated an ECM model by infecting C57BL/6 J mice with the PbA strain. Brain tissues were collected to evaluate Hz deposition and neuronal injury through IF staining and histopathological staining. IF staining revealed a marked reduction in NeuN expression in the cortical neurons of ECM mice, indicative of substantial neuronal loss or dysfunction in the ECM brain (Fig. 1A, B). H&E and Prussian blue staining revealed significant Hz deposition in the perivascular infiltrates of the cerebral cortex in ECM mice (Fig. 1C, D; Fig. S1). These findings suggest that neuronal injury in the cerebral cortex of ECM mice may be associated with Hz deposition.

### pHz induces neuronal injury in vitro

**Fig. 2:**
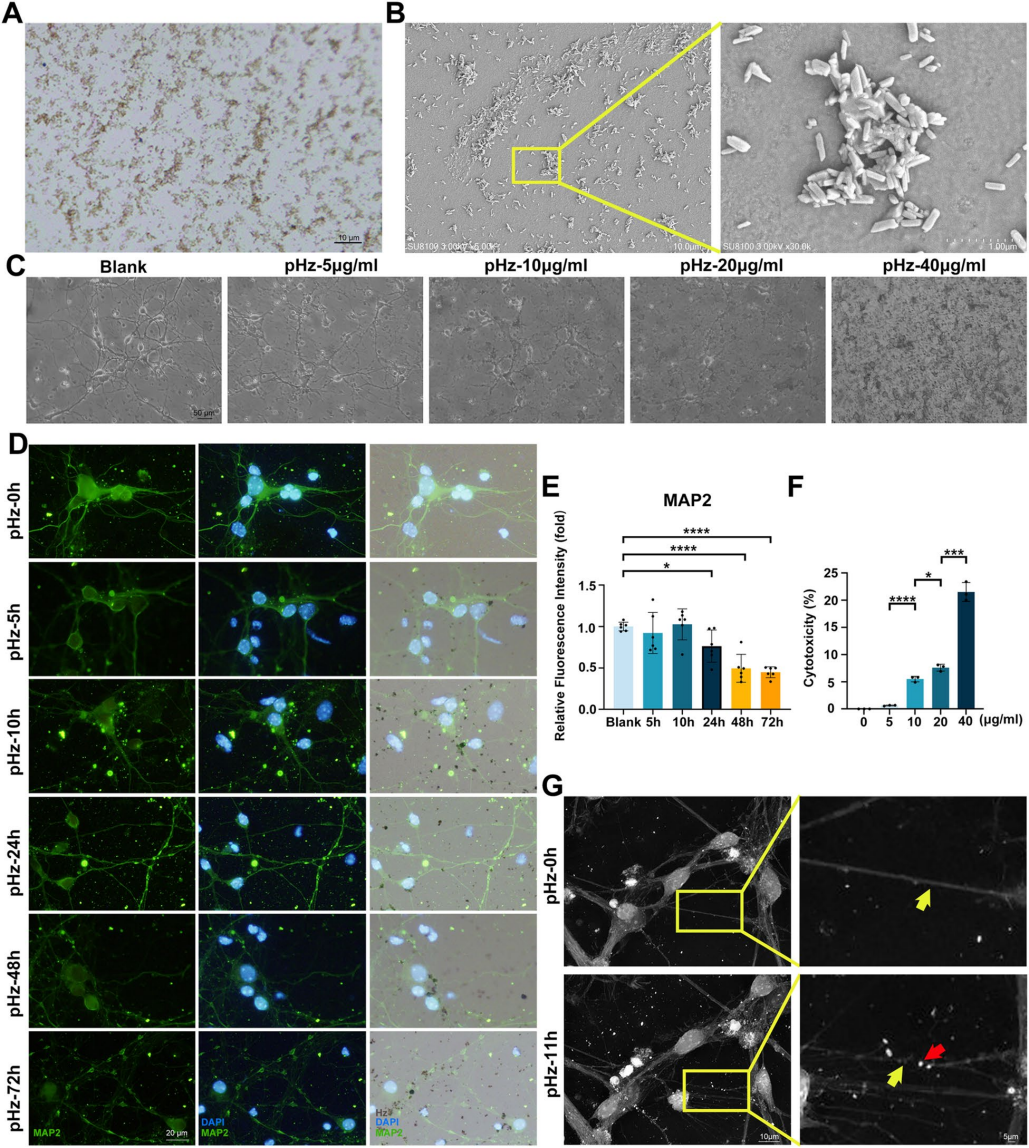
pHz directly induces structural injury to neurons in vitro. **A** Optical microscope observation of pHz. **B** SEM observation of pHz. **C** Optical microscope observation of primary neurons stimulated with graded doses of pHz for 48 h. **D** IF staining of MAP2 in pHz-stimulated primary neurons at different time points. **E** Statistical graph of relative fluorescence intensity of MAP2 in **D**. Data are expressed as mean ± SD; unpaired t-test; *n* = 6 fields per group.** F** LDH relative activity in the supernatant after 48 h of stimulation of primary neurons with graded doses of pHz. Data are expressed as mean ± SD; unpaired t-test; *n* = 3. **G** Live-cell imaging of primary neurons stimulated by pHz at different time points (red arrows: pHz; yellow arrows: neuronal dendrites). *p < 0.05, ***p < 0.001, ****p < 0.0001. pHz, purified hemozoin. SEM, scanning electron microscopy. MAP2, microtubule-associated protein 2. LDH: lactate dehydrogenase

To determine whether Hz directly induces neuronal injury, we extracted and purified Hz in vitro. Morphological characterization using optical microscopy revealed that pHz (purified Hz) appears as uniform brown particles (Fig. 2A). Further observation under SEM demonstrated that pHz exhibits a regularly shaped crystalline structure with dimensions of approximately 0.5 μm (Fig. 2B; Fig. S2), consistent with the size of parasite-derived Hz as reported previously [22]. These pHz particles were subsequently applied to stimulate primary neurons to evaluate its direct neurotoxic effects in vitro.

Following stimulation with graded doses of pHz (5, 10, 20, and 40 μg/ml), optical microscopy revealed that 10 μg/ml pHz was sufficient to induce significant neuronal injury, manifesting as reduced dendritic arborization, axon rupture, and disruption of neuronal network integrity (Fig. 2C). Moreover, IF staining revealed pHz induced time-dependent neuronal injury accompanied by a marked reduction in MAP2 fluorescence intensity (Fig. 2D, E). Specifically, 24-h pHz exposure resulted in observable axonal rupture and a 24% reduction in MAP2 expression. LDH assays also confirmed that​​ there was a dose-dependent exacerbation of neuronal injury​​ following stimulation with graded doses of pHz (Fig. 2F). Furthermore, live-cell imaging​​ demonstrated that pHz ​​adhered to neuronal dendrites​​, ​​resulting in​​ dendritic rupture (Fig. 2G). This direct physical interaction between pHz and neurons suggests that physical adhesion may serve as an initial trigger for structural damage.

Collectively, these results confirm that pHz directly induces neuronal injury, with the severity of injury increasing with pHz dose and stimulation time, although the mechanisms require further elucidation.

### Internalization of pHz by neurons may be a cause of neuronal injury

**Fig. 3:**
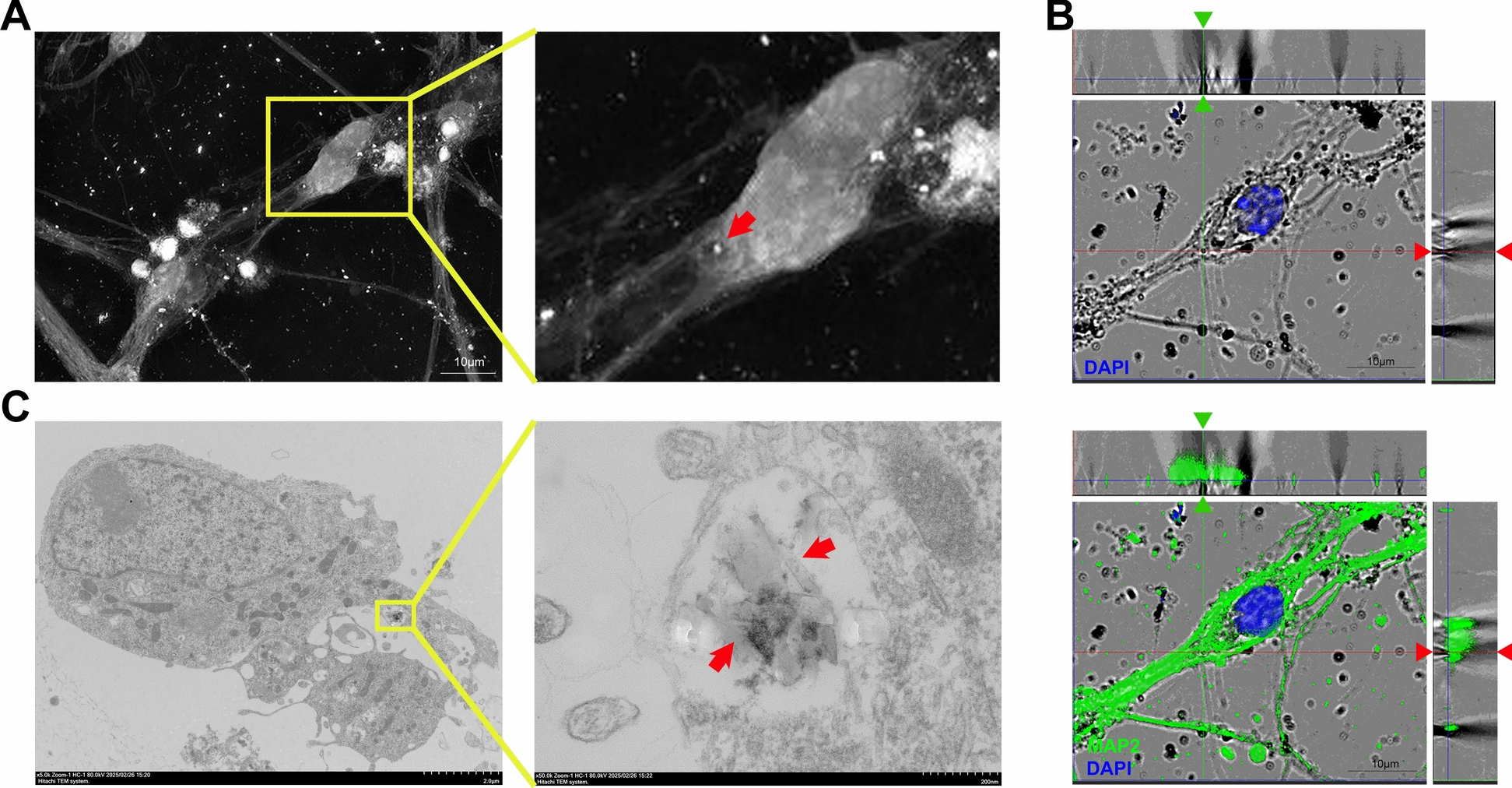
Neurons internalize pHz into the cytoplasm. **A** Live-cell imaging of primary neurons stimulated by pHz at different time points (red arrows: pHz). **B** CLSM-based 3D reconstruction images of primary neurons after 48 h of stimulation with pHz. **C** TEM observation of primary neurons after 48 h of stimulation with pHz (red arrows: pHz). pHz, purified hemozoin. CLSM, confocal laser scanning microscopy. TEM, transmission electron microscopy

To elucidate the mechanism underlying ​​pHz-induced​​ neuronal injury, we tracked the interaction between pHz and primary neurons using multiple imaging techniques. Live-cell imaging was performed on primary neurons following pHz stimulation,​​ revealing that neurons internalized a portion of pHz into the cytoplasm (Fig. 3A; Video S1). This observation was also supported by confocal laser scanning microscopy (CLSM)-based 3D reconstruction, demonstrating pHz encapsulation within MAP2-positive structures across all sectional planes, confirming cytoplasmic localization (Fig. 3B). Subsequent ultrastructural analysis ​​via​​ TEM​​ further confirmed that a portion of pHz was deposited inside neurons. pHz with a regularly shaped crystalline structure was observed in the cytoplasm of neurons, where it was encapsulated within a single-membrane structure (Fig. 3C; Fig. S3).

Collectively, these findings demonstrate that the internalization of pHz by neurons leads to its accumulation within the cytoplasm. This process may represent a critical mechanism underlying neuronal injury.

### pHz induces mitochondrial damage and dysfunction in neurons

**Fig. 4:**
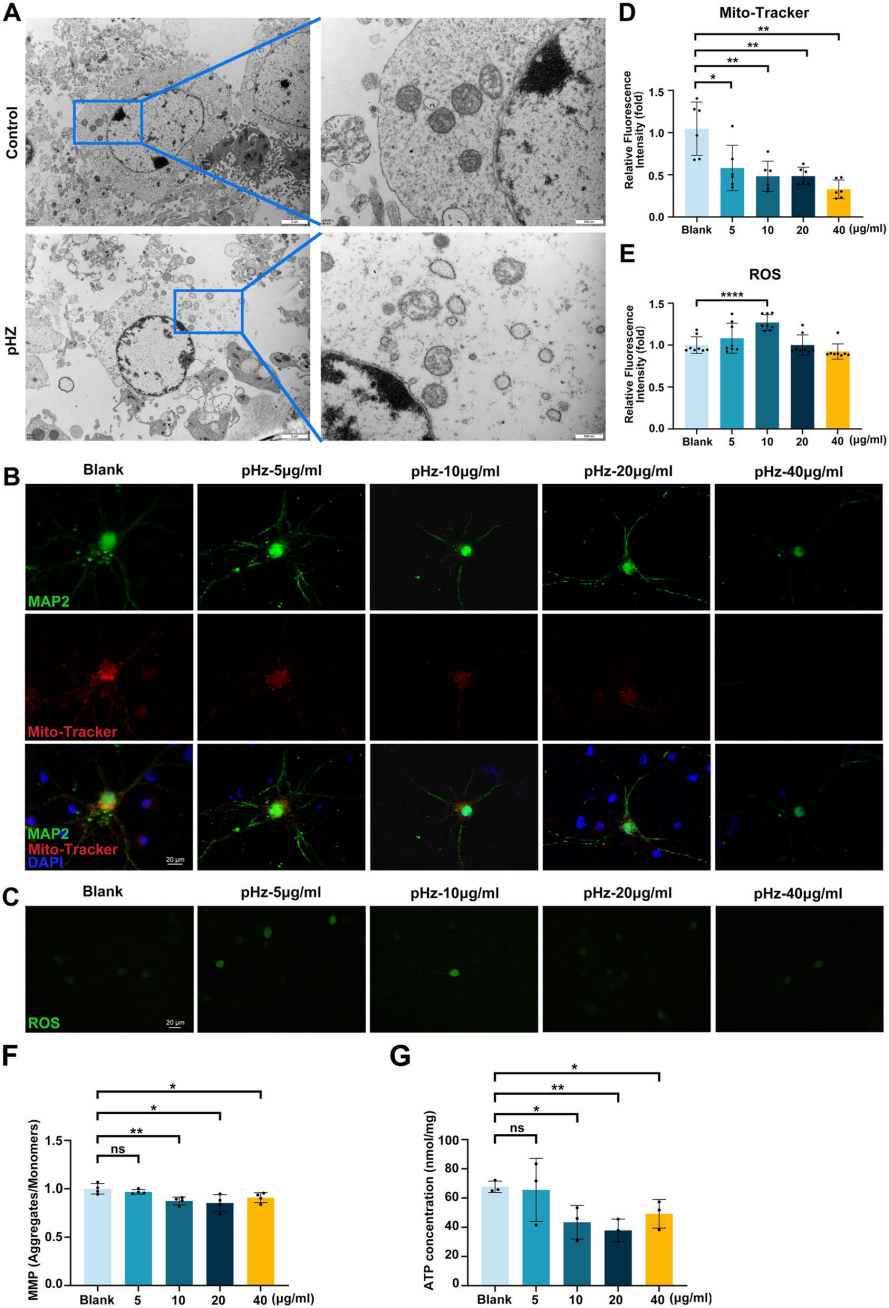
pHz induces mitochondrial structural disruption and dysfunction in neurons. **A** TEM observation of primary neurons after 48 h of stimulation with pHz. **B** IF staining of Mito-tracker in primary neurons after 48 h of stimulation with graded doses of pHz. **C** ROS fluorescent probe staining in primary neurons after 48 h of stimulation with graded doses of pHz. **D** Statistical graph of relative fluorescence intensity of Mito-Tracker in (**B**). Data are expressed as mean ± SD; unpaired t-test; *n* = 6 fields per group. **E** Statistical graph of relative fluorescence intensity of ROS in (**C**). Data are expressed as mean ± SD; unpaired t-test; *n* = 8 fields per group. **F** Statistical graph of MMP (JC-1 ratio) in primary neurons after 48 h of stimulation with graded doses of pHz. Data are expressed as mean ± SD; unpaired t-test; *n* = 4. **G** Statistical graph of ATP content in primary neurons after 48 h of stimulation with graded doses of pHz. Data are expressed as mean ± SD; unpaired t-test; *n* = 3. *p < 0.05, **p < 0.01, ****p < 0.0001, ns, not significant (*p* > 0.05). pHz, purified hemozoin. TEM, transmission electron microscopy. IF, immunofluorescent. ROS, reactive oxygen species. Mito-Tracker, mitochondria tracker. MAP2, microtubule-associated protein 2. JC-1, 5,5′,6,6′-Tetrachloro-1,1′,3,3′-tetraethylbenzimidazolocarbocyanine iodide. ATP, adenosine triphosphate

To investigate the subcellular mechanisms underlying pHz-induced neuronal injury, we focused on mitochondrial integrity and function, as mitochondria are critical for neuronal energy metabolism and survival. TEM analysis of neurons revealed neuronal plasma membrane rupture, mitochondrial shrinkage, and cristae reduction (Fig. 4A). IF staining also demonstrated a dose-dependent reduction in mitochondrial content following stimulation with graded doses of pHz, with fluorescence quantification showing a 52% decrease in mitochondrial fluorescence intensity at 10 μg/ml pHz. These results indicated that neuronal injury induced by pHz was accompanied by a significant depletion of functional mitochondria (Fig. 4B, D). Concurrently, this depletion correlated with elevated reactive oxygen species (ROS) accumulation (Fig. 4C, E). Damaged mitochondria likely initiate ROS surges, which exacerbate oxidative cellular damage and create a vicious cycle of mitochondrial impairment. Mitochondrial function was further assessed via MMP and ATP level measurements, which revealed a significant decline in both MMP and ATP content in neurons. Specifically, 10 μg/ml pHz induced a 13% reduction in MMP and a 36% decrease in ATP content, indicating severe impairment of cellular energy metabolism (Fig. 4F, G).

Collectively, these findings demonstrate that pHz induces a cascade of mitochondrial abnormalities in primary neurons, including structural disruption, reduced mitochondrial content, and dysfunction. These mitochondrial defects collectively contribute to neuronal dysfunction and injury, highlighting mitochondria as a key target of pHz neurotoxicity.

### pHz induces intraneuronal stress responses and neurotransmitter dysregulation

**Fig. 5:**
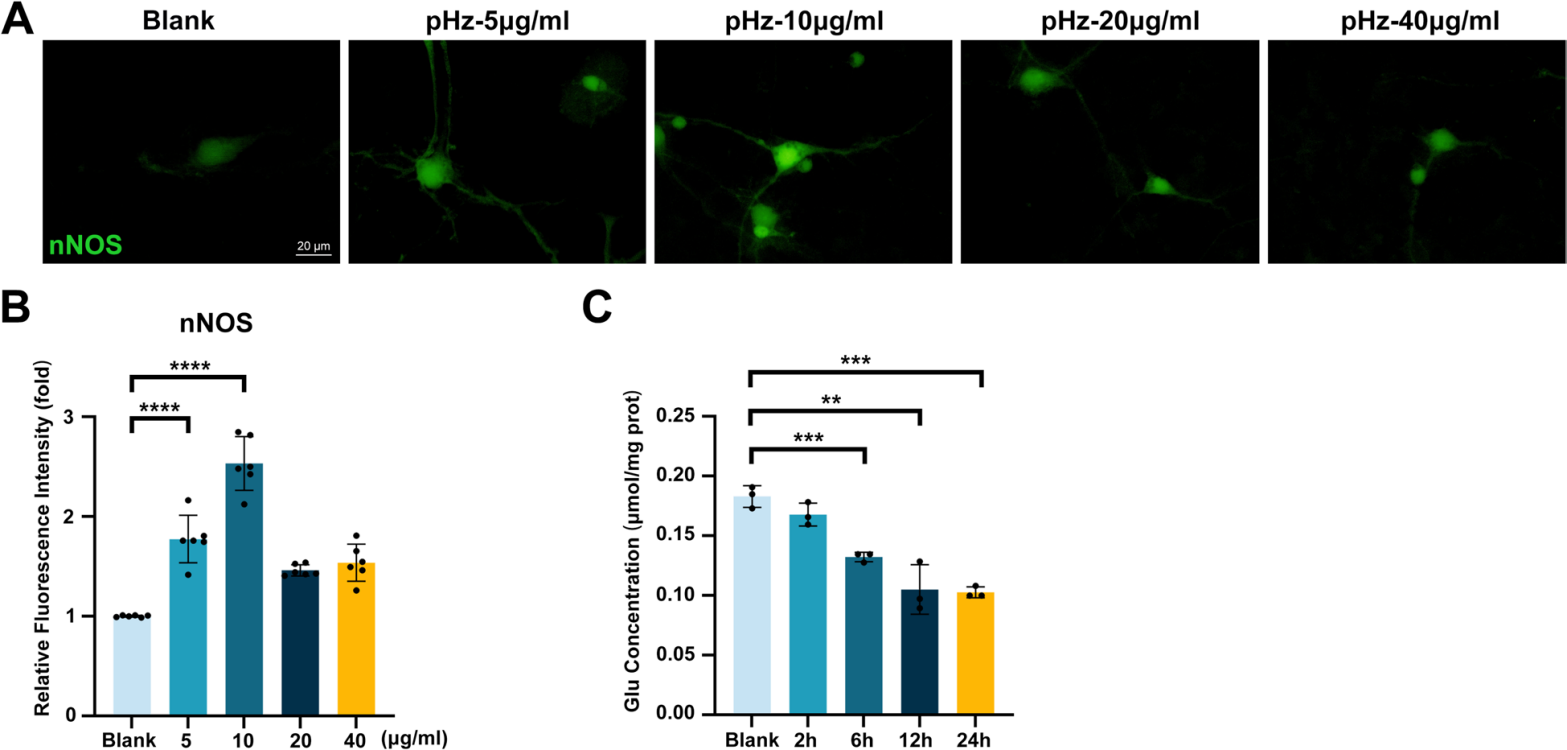
pHz activates neuronal nNOS Upregulation and induces glutamate metabolic dysregulation. **A** nNOS fluorescent probe staining in primary neurons after 48 h of stimulation with graded doses of pHz. **B** Statistical graph of the relative fluorescence intensity of nNOS in (**A**). Data are presented as mean ± SD; unpaired t-test; *n* = 6 fields per group. **C** Glu content in primary neurons stimulated by pHz at different time points. Data are expressed as mean ± SD; unpaired t-test; *n* = 3. **p < 0.01, ***p < 0.001, ****p < 0.0001. pHz: purified hemozoin. nNOS: neuronal nitric oxide synthase. Glu: glutamate

As a key enzyme catalyzing nitric oxide (NO) synthesis, neuronal nitric oxide synthase (nNOS) activity changes reflect the dynamic stress responses of neurons under inflammatory or injured conditions, and its activity is closely associated with neuronal survival, regeneration, and ​​immunopathological damage progression [23, 24]. nNOS activity​​ was ​​significantly upregulated​​ following stimulation with 5 μg/ml and 10 μg/ml pHz. However, no significant increase in nNOS activity was observed after stimulation with 20 μg/ml and 40 μg/ml pHz, potentially attributable to this excessive neuronal injury (Fig. 5​​A, B​​). Additionally, neurotransmitter alterations are sensitive biomarkers of neuronal metabolic function [25]. Glutamate (Glu) levels in neurons were measured at multiple time points (2, 6, 12, and 24 h) following pHz stimulation, which revealed a significant decrease in Glu levels at 6 h. These results indicated a time-dependent decline in Glu levels that correlated with the progressive disruption of neurotransmitter homeostasis during prolonged exposure (Fig. 5C​​).

In conclusion, these findings demonstrate that pHz triggers ​​intraneuronal stress responses and neurotransmitter dysregulation.

## Discussion

CM, a severe complication of malaria, manifests​​ residual neurological sequelae closely associated with neuronal injury [2, 3]. Clinical observations in fatal pediatric CM cases reveal axonal degeneration, with the severity of neuronal injury showing a direct correlation with coma depth [26]. Studies in ECM mouse models demonstrate neurological deficits accompanied by neuronal loss and dendritic spine abnormalities, mediated by molecular pathways such as overexpression of dopamine receptors or dysregulation of the LIMK-1/cofilin-1 pathway [27, 28]. The deposition of Hz, a metabolic byproduct of *Plasmodium*, in the brain parenchyma following BBB disruption has been implicated as a critical pathogenic factor in driving persistent neuronal injury [14, 15]. Elucidating these pathological mechanisms is essential for identifying novel therapeutic targets to enhance adjuvant therapy for CM. In this study, we found that Hz stimulation can cause significant neuronal injury, characterized by reduced dendritic arborization, axon rupture, and plasma membrane disruption, mitochondrial structural damage and dysfunction, as well as inflammatory responses and metabolic dysregulation. Such neuronal injury may stem from either Hz adhesion to neuronal surfaces or its internalization by neurons.

During CM pathogenesis, parasite-derived components released by the rupture of *Plasmodium* parasites are disseminated systemically, enter the disrupted BBB, and accumulate in the brain parenchyma. These processes constitute the core pathological mechanism of CM [4, 29, 30]. We observed significant perivascular Hz deposition in the cerebral cortex of ECM mice. Aligning with existing literature, Ngo-Thanh et al. reported prominent Hz deposition in PbA-infected mouse brains [31]. Therefore, Hz appears to be a key contributor to neuronal injury in CM. Similarly, in malaria-associated acute respiratory distress syndrome (ARDS), Hz accumulates in alveolar and interstitial neutrophils/macrophages. Pulmonary Hz deposition in *Plasmodium berghei* NK65-E-infected mice shows a direct correlation with lung edema, leukocyte infiltration, and inflammatory responses [32–34]. As the primary deposition site for Hz [35], hepatic Hz levels in *Plasmodium chabaudi*-infected mice are associated with enzyme leakage and lipid peroxidation [36]. During placental malaria (PM), Hz localizes to the intervillous fibrin deposits and infected cells, triggering persistent inflammation and trophoblast damage [37, 38]. Collectively, Hz deposition in severe malaria patients is strongly correlated with clinical complications.

Neuronal injury is​​ the primary cause of neurological sequelae in CM patients, ​​yet​​ its pathogenesis remains incompletely understood ​​[39]. Previous studies indicate that neuroinflammation, oxidative stress, ​​and​​ immune cell-derived inflammatory mediators contribute to neuronal death and synaptic dysfunction [7, 40]. ​​Furthermore, our previous research demonstrated that activated CD8⁺ T cells infiltrate the brain parenchyma and play a significant role in neuronal injury via cytotoxic effects and ferroptosis induction [11, 12, 41].​​ Here, we observed that Hz adheres to neuronal dendrites and axons, resulting in reduced dendritic arborization and axon rupture. This physical adhesion likely acts as the initial trigger for structural neuronal injury. Although prior studies have linked Hz to neuronal apoptosis, the specific mechanisms remain unclear. Our findings further demonstrated that neurons internalize Hz, which might trigger inflammatory cascades and neuronal dysfunction, culminating in neuronal injury.

This study first observed that neurons internalize Hz, which elicits cellular injury. However, the molecular mechanisms of Hz neurotoxicity remain incompletely elucidated. Raulf et al. showed that C-type lectin receptor 12 A (CLEC12A) specifically recognizes Hz, thereby enhancing ​​the​​ cross-activation of CD8⁺ T cells by dendritic cells. Notably, this study did not directly validate the binding of Hz to CLEC12A under physiological conditions [42]. Hz can activate monocytes via the ​​T​​oll-​​L​​ike ​​R​​eceptor ​​4​ (TLR-4) and CD11b/CD18 integrin pathways after binding to host fibrinogen [43]. Furthermore, Hz can ​​serve​​ as a non-classical ligand for TLR9 and ​​activate​​ innate immunity through a myeloid differentiation primary response 88 (MyD88)-dependent pathway [44]. However, alternative views suggest that this activation might stem from hemozoin-bound DNA [45]. Peroxisome proliferator-activated receptor γ (PPARγ) is an important transcription factor in the brain. Hz can upregulate PPARγ mRNA expression in dendritic cells (DCs) [46]. In studies on multiple sclerosis (MS), excessive activation of PPARγ can inhibit mitophagy and exacerbate the accumulation of ROS and oxidative stress [47]. Whether Hz-induced neuronal injury in the present study is associated with the activation of PPARγ warrants further investigation. More recently, a study using human induced pluripotent stem cell (iPSC)-​​differentiated​​ neuronal models revealed that Hz triggers neuroinflammation and contributes to CM through molecular pathways involving activation of the DNA damage response, the p38 MAPK signaling pathway, and neurodegenerative ​​disease-associated​​ pathways [48]. Similarly, monosodium urate (MSU) crystals, which are common pathogenic uric acid crystals, have been implicated in studies where TLR2/TLR4 mediate their recognition, while others have suggested that MSU is opsonized by complement/antibodies for phagocytosis by macrophages, ultimately ​​promoting​​ gout inflammation [49–51]. Despite these advancements, the specific molecular mechanisms of Hz-induced neuronal injury await full clarification.

Oxidative stress and lipoperoxidation play important roles in malaria pathogenesis [52, 53], and Hz is a key factor inducing the upregulation of ROS and lipoperoxidation. Studies have shown that both natural hemozoin (nHz) and sHz induce a concentration-dependent increase in ROS levels within macrophages [54]. In PM, Hz induces ROS production and lipid peroxidation in primary human syncytiotrophoblasts (STs), and it generates toxic products such as 4-hydroxynonenal (4-HNE) [55]. Cytochrome P450 enzymes (CYPs) are key mediators of ROS generation and oxidative stress, with their catalytic activity significantly elevated in the brain, kidney, and testis of rats, which is highly dependent on heme [56, 57]. However, whether the upregulation of ROS induced by Hz is associated with CYPs warrants further investigation, although Hz has a structure similar to heme. This study confirmed that Hz stimulation induced significant ROS production in neurons, accompanied by abnormal mitochondrial morphology, diminished MMP, and reduced mitochondrial content. Gastrodin and isoliquiritigenin effectively alleviate oxidative stress and neuronal injury by enhancing antioxidant enzyme expression in Parkinson's disease and cerebral ischemia models [58, 59]. Similarly, targeted ROS scavenging or inhibition of their production may become a potential therapeutic strategy for neuronal injury in ECM, but specific research in this field remains to be conducted.

In conclusion, our study elucidated the pathogenic mechanism through which Hz directly induces neuronal injury through physical adhesion and neuronal internalization. This finding suggests that Hz-mediated neuronal injury could be ​​a potential trigger for neurological sequelae in CM survivors and provides a theoretical basis for the development of ​​adjunctive​​ therapies targeting Hz. However, the specific molecular pathways and ​​cellular regulatory networks still ​​require further clarification.

## Conclusions

This study ​​demonstrates​​ that Hz represents a pivotal pathogenic factor in ECM-associated neuronal injury, eliciting structural damage characterized by​​ reduced dendritic arborization, axon rupture, and plasma membrane disruption through physical adhesion and internalization. ​​Furthermore​​, Hz ​​induces​​ reduced mitochondrial content, mitochondrial structural damage and dysfunction, as well as intraneuronal stress responses and neurotransmitter dysregulation. These findings establish Hz as a key effector of neuronal injury in ECM, laying a mechanistic foundation for developing adjuvant therapies targeting Hz to alleviate neurological sequelae.

## Supplementary Information

Supplementary Material 1: Video. S1. Live-cell imaging of primary neurons stimulated by pHz at different time points (red circle: pHz).

Supplementary Material 2: Fig. S1. H&E staining of cerebral cortical tissue sections from ECM mice (green arrows: Hz).

Supplementary Material 3: Fig. S2. Chemical molecular structure of hemozoin (drawn with ChemDraw 2022).

Supplementary Material 4: Fig. S3. Transmission electron microscopy observation of primary neurons after 48 h of stimulation with pHz (red arrows: pHz).

## Data Availability

Data supporting the main conclusions of this study are included in the manuscript.

## Abbreviations

ARDS: Acute respiratory distress syndrome
CM: Cerebral malaria
CLSM: Confocal laser scanning microscopy
CYPs: Cytochrome P450 enzymes
CLEC12A: C-type lectin receptor 12 A
DCs: Dendritic cells
ECM: Experimental cerebral malaria
FBS: Fetal bovine serum
Glu: Glutamate
H&E: Hematoxylin and eosin
HBSS: Hank’s balanced salt solution
HIER: Heat-induced epitope retrieval
Hz: Hemozoin
IF: Immunofluorescent
iPSC: Induced pluripotent stem cell
LDH: Lactate dehydrogenase
MAP2: Microtubule-associated protein 2
MMP: Mitochondrial membrane potential
MyD88: Myeloid differentiation primary response 88
MSU: Monosodium urate
MS: Multiple sclerosis
MDA: Malondialdehyde
NeuN: Neuronal nuclei
NO: Nitric oxide
nNOS: Neuronal nitric oxide synthase
nHz: Natural hemozoin
PbA: *Plasmodium Berghei* ANKA
pHz: Purified hemozoin
PM: Placental malaria
PPARγ: Peroxisome proliferator-activated receptor γ
ROS: Reactive oxygen species
sHz: Synthetic hemozoin
SMA: Severe malarial anemia
SPF: Specific pathogen-free
SEM: Scanning electron microscopy
STs: Syncytiotrophoblasts
TEM: Transmission electron microscopy
TLR-4: Toll-like receptor 4
4-HNE: 4-Hydroxynonenal
