# Supplementary figures and images for: Hemozoin induces neuronal injury primarily characterized by axon rupture and mitochondrial damage in experimental cerebral malaria
Source: Parasit Vectors. 2025 Dec 18;19:43. doi: 10.1186/s13071-025-07102-5 (PMC12829028; doi:10.1186/s13071-025-07102-5)

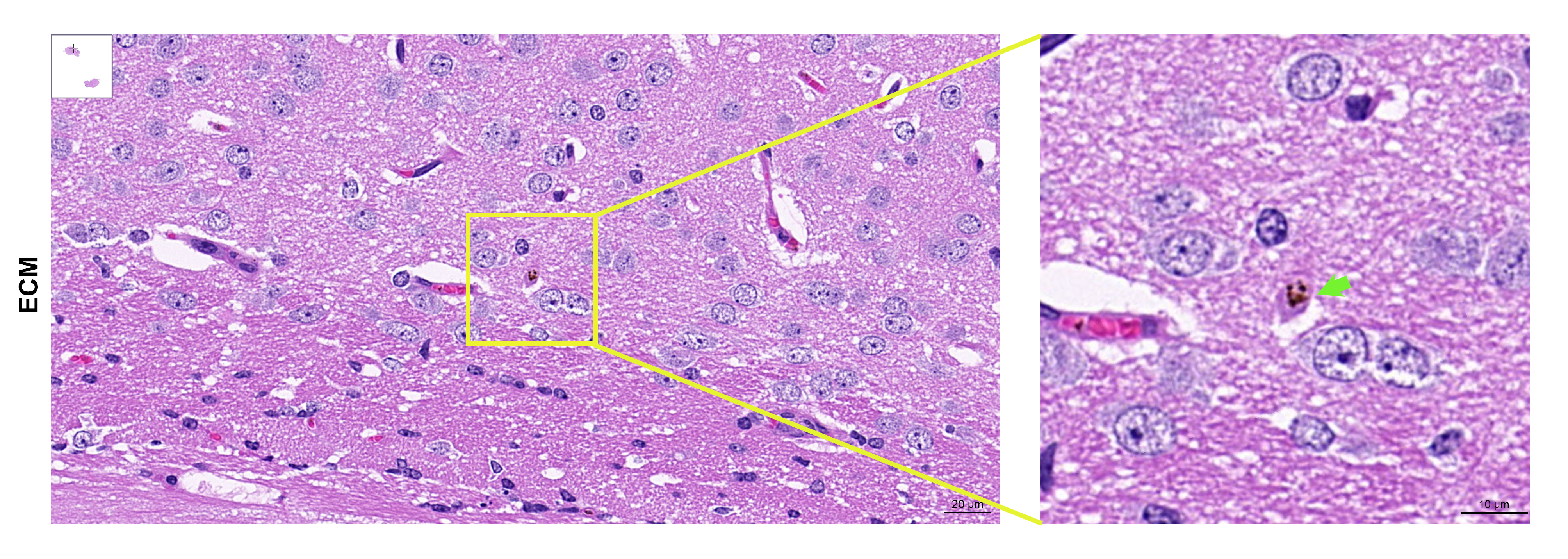

Supplement: Supplementary file 2 — Supplementary Material 2: Fig. S1. H&E staining of cerebral cortical tissue sections from ECM mice (green arrows: Hz). [file 13071_2025_7102_MOESM2_ESM.tif]

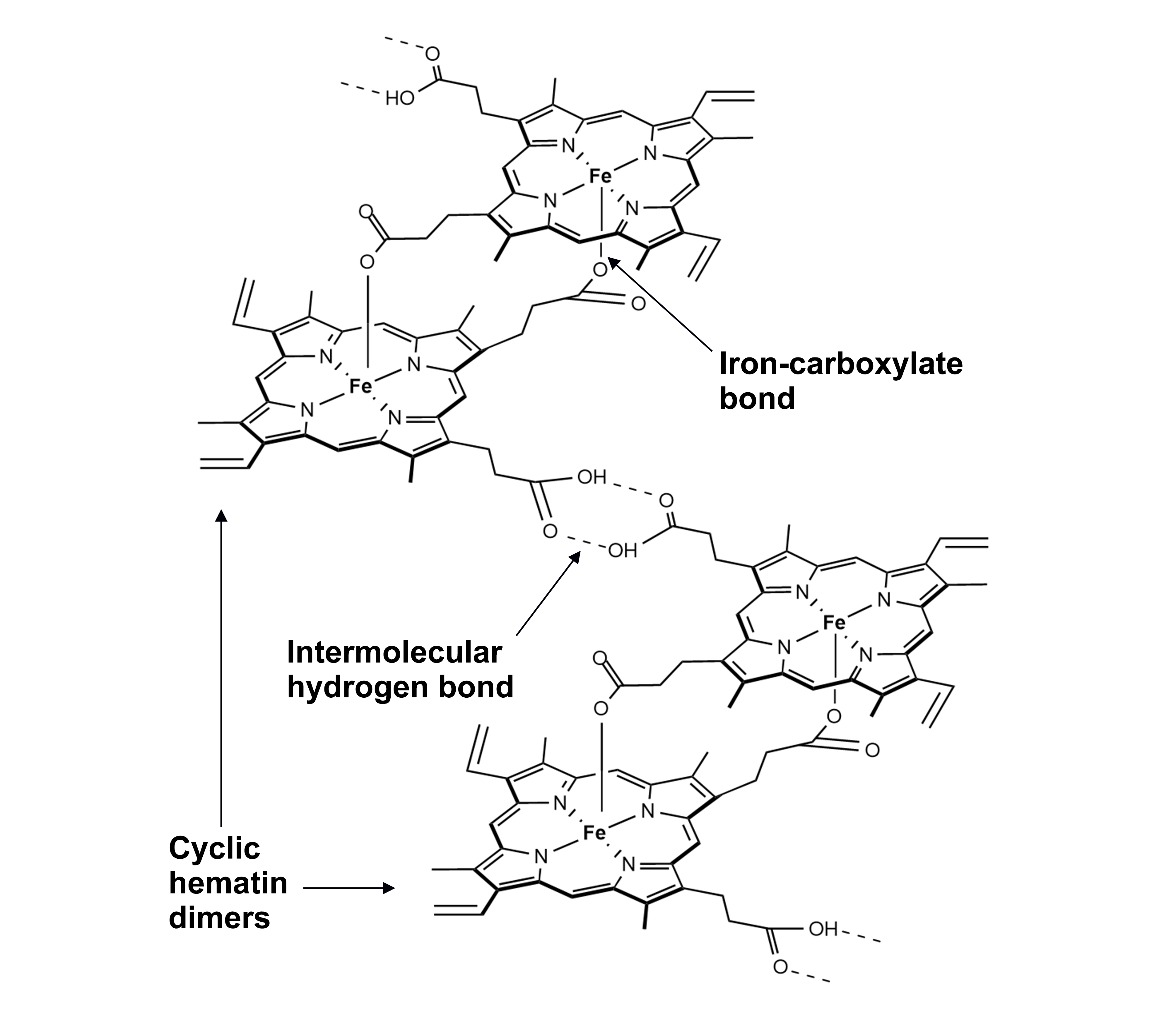

Supplement: Supplementary file 3 — Supplementary Material 3: Fig. S2. Chemical molecular structure of hemozoin (drawn with ChemDraw 2022). [file 13071_2025_7102_MOESM3_ESM.tif]

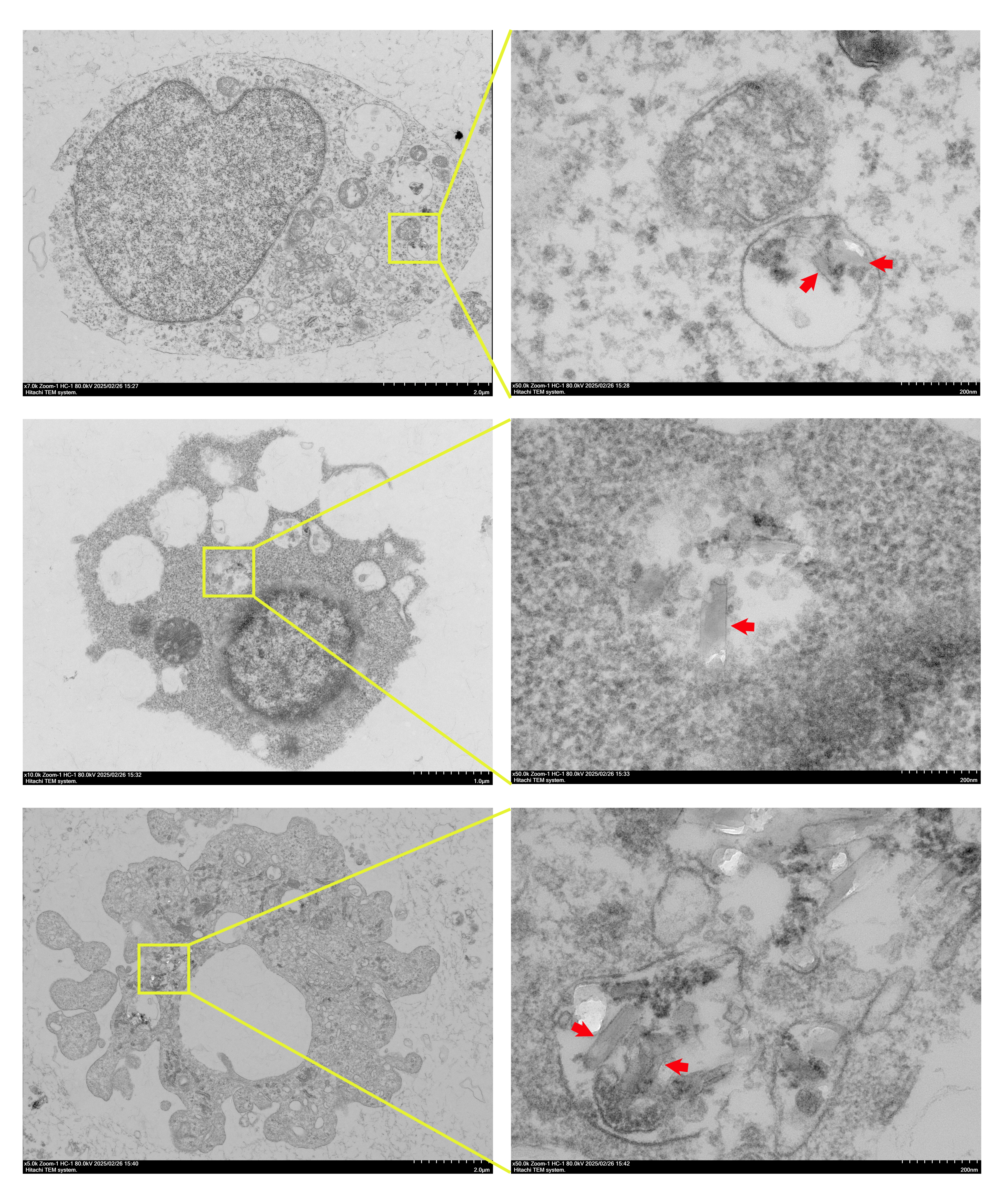

Supplement: Supplementary file 4 — Supplementary Material 4: Fig. S3. Transmission electron microscopy observation of primary neurons after 48 h of stimulation with pHz (red arrows: pHz). [file 13071_2025_7102_MOESM4_ESM.tif]
